# Improving the Performance of Zn-Air Batteries with N-Doped Electroexfoliated Graphene

**DOI:** 10.3390/ma13092115

**Published:** 2020-05-02

**Authors:** Anna Ilnicka, Malgorzata Skorupska, Piotr Romanowski, Piotr Kamedulski, Jerzy P. Lukaszewicz

**Affiliations:** 1 Faculty of Chemistry, Nicolaus Copernicus University in Torun, Gagarina 7, 87-100 Torun, Poland; ailnicka@umk.pl (A.I.); m.skorupska@doktorant.umk.pl (M.S.); 285492@stud.umk.pl (P.R.); pkamedulski@umk.pl (P.K.); 2 Centre for Modern Interdisciplinary Technologies, Nicolaus Copernicus University in Torun, Wilenska 4, 87-100 Torun, Poland

**Keywords:** FL-graphene, exfoliation, nitrogen-doped structure, oxygen reduction reaction, rechargeable Zn-air battery

## Abstract

The constantly growing demand for active, durable, and low-cost electrocatalysts usable in energy storage devices, such as supercapacitors or electrodes in metal-air batteries, has triggered the rapid development of heteroatom-doped carbon materials, which would, among other things, exhibit high catalytic activity in the oxygen reduction reaction (ORR). In this article, a method of synthesizing nitrogen-doped graphene is proposed. Few-layered graphene sheets (FL-graphene) were prepared by electrochemical exfoliation of commercial graphite in a Na_2_SO_4_ electrolyte with added calcium carbonate as a separator of newly-exfoliated FL-graphene sheets. Exfoliated FL-graphene was impregnated with a suspension of green algae used as a nitrogen carrier. Impregnated FL-graphene was carbonized at a high temperature under the flow of nitrogen. The N-doped FL-graphene was characterized through instrumental methods: high-resolution transmission electron microscopy, X-ray photoelectron spectroscopy, and Raman spectroscopy. Electrochemical performance was determined using cyclic voltamperometry and linear sweep voltamperometry to check catalytic activity in ORR. The N-doped electroexfoliated FL-graphene obeyed the four-electron transfer pathways, leading us to further test these materials as electrode components in rechargeable zinc-air batteries. The obtained results for Zn-air batteries are very important for future development of industry, because the proposed graphene electrode materials do not contain any heavy and noble metals in their composition.

## 1. Introduction

The oxygen reduction reaction (ORR) is important in many energy conversion and storage technologies, including fuel cells, metal-air batteries, and solar-based energy harvesting systems [1,2]. Today’s applied energy storage technology is dominated by Li-based batteries, typically exploiting intercalation technology. However, their real energy density is limited to <350–400 Wh kg^−1^ [3,4]. High cost is another drawback. Metal-air batteries have commonly been regarded as the most serious competitor to lithium intercalation battery technology for some time. Li-air batteries are presumed to have a theoretical energy density of >1000 Wh kg^−1^. Li–air batteries provide a specific energy of ~6.12 MJ/kg at the cell level, which is ca. 4–5 times greater compared to a commercial lithium-ion battery [3]. The demand for lithium and its derivatives is constantly growing, recently raising concerns of future lithium accessibility. Metallic lithium is dangerous in practical handling. Conversely, zinc is a much more common and less dangerous element, whose accessibility and prices are not expected to change dramatically in the coming decades. Thus, zinc-based technology of electrochemical energy accumulation seems to be a reasonable alternative to all Li-based solutions. This conclusion is greatly strengthened through the analysis of the electrochemical parameters of Zn-batteries [4]. Many reviews compare different high-energy, metal-air batteries (Zn-air, Li-air, and Na-air batteries) and present the advantages and disadvantages of this battery type. Despite numerous advances in research on this subject, many problems persist, limiting the capabilities of metal-air batteries [5,6]. The biggest advantage of these batteries, especially Zn-air batteries, is their low-cost production. Though Zn-air batteries have less energy than Li-air batteries (theoretical energy density for Li-air batteries: 3500 Wh kg^−1^ [7,8], for Zn-air: 1084 Wh kg^−1^ [9,10,11]), they are safer devices to produce. This kind of battery has a lot of future potential for energy storage devices. However, the effectiveness of a metal-air battery depends on the cathode’s ability to perform four-electron oxygen reduction reactions (ORR), for which it is essential to synthesize appropriate electrocatalysts. As of today, known effective electrocatalysts are based on expensive and/or environment-unfriendly components, like platinum [12,13] and ruthenium [14]. So far, there are reports of noble-metal-free catalysts for ORR based on alloys [15,16], nitrides [17,18], perovskite oxides [19], spinel oxides [20], manganese sulfides [21], mesoporous Ni/NiO nanosheets [22], and nitrogen-doped metal sulfides [23]. At the same time, researchers have long been attempting to find alternative carbon-based catalysts [24,25,26,27,28,29]. Carbon materials that have been successfully used as catalysts in ORR include graphene [30,31,32], graphene quantum dots (GQDs) [33], carbon nanotubes (CNTs) [34,35,36,37,38,39], 3D carbon nanoframes [40], and carbon nanofibers [26,41,42]. Compared to CNTs, graphene, pyrolyzed metal-organic frameworks, and carbon fibers have much wider applications, thanks to their high electrical and thermal conductivity, large surface area, and stable chemical properties [43,44,45]. Doping these materials with heteroatoms such as nitrogen, boron, phosphorus, or sulfur can increase their catalytic activity towards ORR [30,46]. This sort of heteroatom doping of carbon materials may be a way to improve electric conductivity and electrochemical efficiency, where surface functional groups play a crucial role [47,48]. Developing carbon-based metal-free functional catalysts (particularly for ORR) is still highly challenging; several announcements were recently published, including the paper by Zhang et al., describing nitrogen and phosphorus co-doping of 3D-structured graphene oxide [49]. It remains a substantial challenge to develop low-cost, heteroatom-doped carbon catalysts, which would exhibit outstanding electrocatalytic activity and electrochemical stability.

Herein, the authors report the synthesis of a series of highly-efficient N-doped graphene-based electrocatalysts. The catalysts were synthesized for the first time via the thermal degradation of green *Chlorella vulgaris* algae among electroexfoliated graphene sheets (FL-graphene). The obtained metal-free N-doped graphene exhibited outstanding activity in catalyzing ORR. Moreover, the series of synthetized graphene catalysts were tested as the air cathode in a home-made rechargeable Zn-air battery, demonstrating excellent activity and stability.

## 2. Materials and Methods 

### 2.1. Preparation of N-Doped Graphene

N-doped graphene was synthesized in two steps. In the first step, graphene sheets were acquired by means of electrochemical exfoliation of extra-pure graphite in a home-made experimental setup. The process of graphite powder electroexfoliation was described in our previous paper [50]. The cathode and anode were made of platinum: a piece of mesh (top electrode—anode) and a round plate (bottom electrode—cathode). The graphite powder, dispersed in an aqueous electrolyte, was in permanent contact with the electrodes. Additionally, a separator (nanopowder of calcium carbonate) was premixed with graphite in a mass ratio of 2:1. The powdered graphite and separator were dispersed in 10 mL 1 M water solution of Na_2_SO_4_. A polymer membrane, which was permeable only for electrolyte ions, made it possible to separate cathode and anode zones, and keep the graphite/separator particles near one electrode, but not the other. Direct current was set between the electrodes to trigger electrode reactions of Na^+^ and SO_4_^2−^ ions. Both electrode processes produce gas products: hydrogen at the cathode and oxygen at the anode. Simultaneously, graphite grains were separated into FL-graphene sheets upon the action of gas being evolved at an electrode (hydrogen or oxygen). Graphite grains, a highly conductive material, were frequently in contact with one of the platinum electrodes (anode or cathode), and the electrode potential extended onto their bulk and surface. Thus, graphite particles worked in a way similar to that of the platinum surface, i.e., gas evolved at the graphite, enabling its splitting into less associated graphene structures (mainly FL-graphene). In the first step of this study, graphite was subjected to anodic exfoliation, after which each sample was sonicated for 30 min, then washed with distilled water. This process was repeated twice. Finally, the samples were dried overnight in an oven at 80 °C. In the second step, the graphene produced through electroexfoliation in step one was enriched with nitrogen atoms. Exfoliated graphene was impregnated with N-reagent, i.e., a suspension 0.3 g of *Chlorella vulgaris* in 10 mL of ethanol, which was added to exfoliated graphene and sonicated for 30 min in an ultrasonic bath. After that, the resulting mixture was left on a magnetic stirrer to homogenize for 48 h until the ethanol evaporated. The dry residue was carbonized at 800 °C, 850 °C, and 900 °C under the flow of nitrogen and with a heating rate of 10 °C min^−1^. After carbonization, the graphene powder was treated for 20 min with hydrochloric acid (HCl, 36%–38%) to remove the separator. Each sample was then rinsed with distilled water, filtrated, and washed with distilled water again. The obtained samples are designated further as GX, where X refers to the carbonization temperature 800 °C, 850 °C, 900 °C.

### 2.2. Physicochemical Characterization

The atomic structure of the samples was observed by a high-resolution transmission electron microscope (HRTEM FEI Tecnai F20 X-Twin, Brno, Czech Republic) at an accelerating voltage of 200 kV. Raman spectra were obtained by a Renishaw InVia Raman analyzer (laser wavelength 532 nm, Renishaw Company, Gloucestershire, UK). X-ray photoelectron spectroscopy (XPS, PHI5000 VersaProbe II Scanning XPS Microprobe, Chigasaki, Japan) measurements were performed using a monochromatic Al Kα x-ray source. Survey spectra were recorded for all samples in the energy range of 0 to 1300 eV with a 0.5 eV step, high-resolution spectra were recorded with a 0.1 eV step.

### 2.3. Electrochemical Measurements

The ORR catalytic activity was electrochemically evaluated with a rotating disc electrode (RDE). Electrochemical measurements were executed using an Autolab electrochemical workstation (PGSTAT128N, Utrecht, The Netherlands) with a standard three-electrode system in an electrolyte solution of 0.1 M KOH at room temperature (KOH was purchased from POCH, KOH content min. 85%). A glassy carbon electrode (GCE, 5 mm diameter), an Ag/AgCl electrode in 3 M KCl, and Pt wire were used as a working electrode, reference electrode, and counter electrode respectively. A commercial platinum-on-graphitized-carbon Pt/C (20 wt.% Pt) catalyst was acquired from Sigma-Aldrich. 2.5 mg of the catalyst was suspended in 0.4 mL of distilled water, ethanol (1:4), and Nafion (0.5 wt.% Nafion) to form a homogeneous ink through ultrasonication for 60 min. Afterwards, 15.63 μL of the catalyst ink was dropped using a pipette onto the pre-polished glassy carbon electrode (GCE) and dried. The catalyst loading amount was approximately 0.4 mg cm^−2^. The ORR catalytic activity of the samples was evaluated via cyclic voltammetry (CV) at a scan rate of 10 mV s^−1^, as well as linear sweep voltammetry (LSV) at a scan rate of 5 mV s^−1^, in an electrolyte solution of 0.1 M KOH, using an RDE system (Metrohm, Utrecht, the Netherland). The LSV curves were obtained at a rotation speed of 1600 rpm in an oxygen-saturated electrolyte solution. All the experiments were conducted at room temperature. The number of electrons (*n*) involved in the ORR can be deduced from the Koutecky-Levich (K-L) equation:

J^−1^ = J_L_^−1^ + J_K_^−1^ = (Bω^1/2^)^−1^ + J_K_^−1^ (1)

B = 0.62nFC_0_(D_0_)^2/3^ν^−1/6^ (2)

where, J, J_L_, and J_K_ are current densities—measured, diffusion limiting, and kinetic; ω is the angular velocity of the disc; n is the number of transferred electrons in the reaction; F is the Faraday constant (96,485 C mol^−1^); C_0_ is the concentration of dissolved oxygen in 0.1 M KOH (1.2 × 10^−6^ mol L^−1^); D_0_ is the diffusion coefficient of dissolved oxygen in 0.1 M KOH (1.9 × 10^−5^ cm^2^ s^−1^); ν is the kinetic viscosity of 0.1 M KOH (0.01 cm^2^ s^−1^) [51]. According to Equations (1) and (2), n can be obtained from the slope of the K-L plot. All ORR currents presented in the figures are Faradaic currents, after correction for the capacitive current. For Tafel plots, the kinetic current was determined after mass-transport correction of RDE curves by:

J_K_ = (J × J_L_)/(J_L_ − J) (3)

### 2.4. Manufacturing a Zn-Air Battery

A complete Zn-air battery characterization, including the investigation of air electrodes, was performed using a home-made electrochemical setup. The Zn-air battery was assembled from a zinc plate (anode) and nitrogen-doped graphene sheets stuck to a sheet of carbon paper (air-permeable cathode). The air cathode (N-doped graphene) for Zn-air batteries consists of a gas diffusion layer (GDL) in contact with air and a catalytic layer (CL) on the solution side. The carbon paper and a microporous membrane (25 µm thickness polypropylene membrane, Celgard 5550) were used as a separator. A total of 10 mg of the graphene catalyst was dispersed in 0.9 mL of a solution of isopropanol and Nafion (5 wt.%, Sigma Aldrich, Warsaw, Poland) to form a homogeneous ink, which was then sonicated for 1 h. The catalyst ink was loaded onto carbon paper sized at about 2.5 cm × 2.5 cm (loading 1 mg cm^−2^) and dried at 80 °C for 30 min. A piece of zinc plate was used as the anode and 6 M KOH containing 0.2 M ZnCl_2_ (in ratio 9:1) was used as the electrolyte. The Zn-air batteries were tested in ambient conditions. The polarization curves were obtained using the LSV technique with an Autolab electrochemical working station (PGSTAT302N, Utrecht, The Netherland) at 5 mV s^−1^. The galvanostatic discharge and charge cycling (5 min discharge and 5 min charge with the current density of 1 mA cm^−2^) were performed in the Nova testing system. For comparison, a Zn-air battery with Pt/C (20 wt.% Pt, Sigma-Aldrich) as the air electrode catalyst was also tested. The voltage of discharge was 0.9 V. The cycling lasted for 42 h (600 charge/discharge cycles). LSV was measured using a real battery setup to evaluate practical cathode performance, rather than the catalyst activities. The discharge polarization and power density profiles were conducted by a galvanodynamic method with a current density ranging from 0 to 200 mA cm^−2^.

## 3. Results and Discussion

### 3.1. Material Characterization

**Figure 1:**
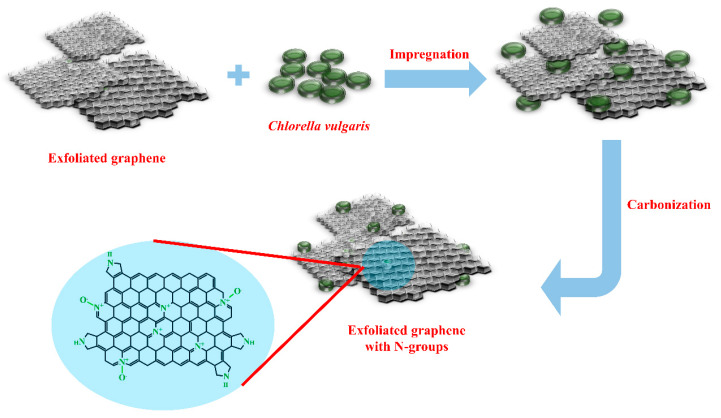
Preparation procedure of N-doped exfoliated graphene synthesis involving electroexfoliation of graphite and *Chlorella vulgaris* as the nitrogen source.

A schematic procedure of the proposed method of exfoliating graphite powder to graphene and introducing heteroatoms into the graphene structure is presented in Figure 1.

The aim of the procedure was to synthesize nitrogen-doped graphene without heavy/noble metals. The key innovation of the study was to eliminate these metals from graphene-type catalysts applied in metal-air batteries, particularly Zn-air ones. The employed electroexfoliation, coupled with the application of a nanoparticle separator, led to 3D structuring of graphene flakes (mainly FL-graphene), as shown in other studies [50]. Durable 3D structuring of separated graphene sheets requires a kind of carbon glue, i.e., an in situ created carbon matrix sticking together and fixing the sheets permanently. In some previous studies, the authors applied furfuryl alcohol (FA) for this purpose. When FA is in the presence of an inorganic acid and the temperature is increased, FA is the first to polymerize (converts into poly(furfuryl alcohol), PFA). Then, PFA decomposes and becomes a source of carbon material, which fixes separated graphene sheets. In the current study, *Chlorella vulgaris* replaced FA/PFA, as depicted in Figure 1.

According to our previous studies and the literature description, nitrogen-containing functional groups on the carbon surface may play the role of effective reaction centers in ORR [52,53,54,55]. During the performed impregnation process, the spaces between exfoliated graphene sheets were filled by *Chlorella vulgaris* cells. *Chlorella vulgaris* is a green microorganism (algae) rich in nitrogen which itself can be successfully converted into a porous carbon matrix with a high nitrogen content (up to 9.83 wt.%) upon carbonization of its dry powder at elevated temperatures [56]. Thus, *Chlorella vulgaris* is a proven nitrogen source that can be exploited during different carbon material manufacturing scenarios. Similarly, in the next step of our study the green algae were efficiently converted into a nitrogen-rich carbon matrix, becoming a part of the whole graphene-based 3D structure. Therefore, nitrogen atoms were present in the manufactured graphene catalysts, i.e., introduced into the structure, with the goal of being ORR centers. 

**Figure 2:**
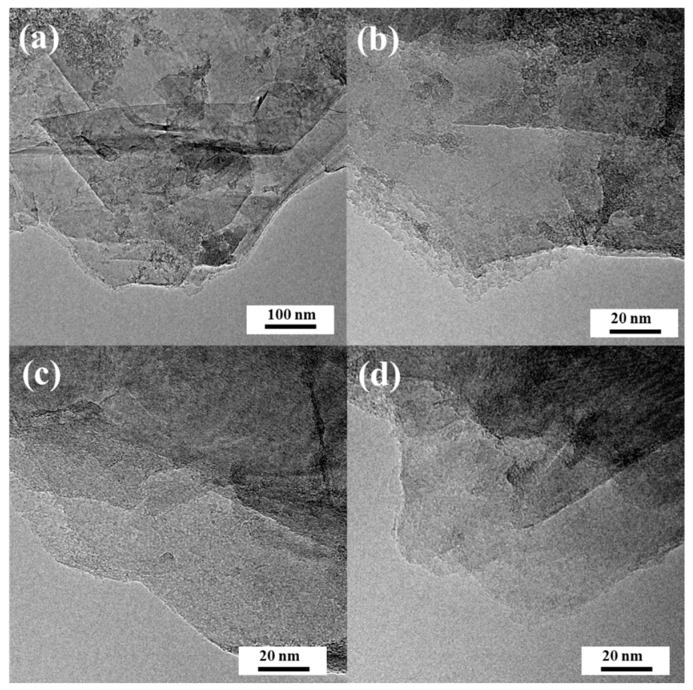
High-resolution transmission electron microscope (HRTEM) images of samples: (**a**,**b**) G800 at different magnifications; (**c**) G850; (**d**) G900.

High-resolution transmission electron microscopy images are presented in Figure 2 and document the electroexfoliation of pristine graphite. For all samples carbonized at different temperatures, G800, G850, and G900, locally stacked parallel layers of graphene are observable, as is typical for graphene-based materials. Graphene sheets in all samples were very thin but evidently visible. The acquired materials reveal overlapping and disordered morphology of the graphene sheets.

**Figure 3:**
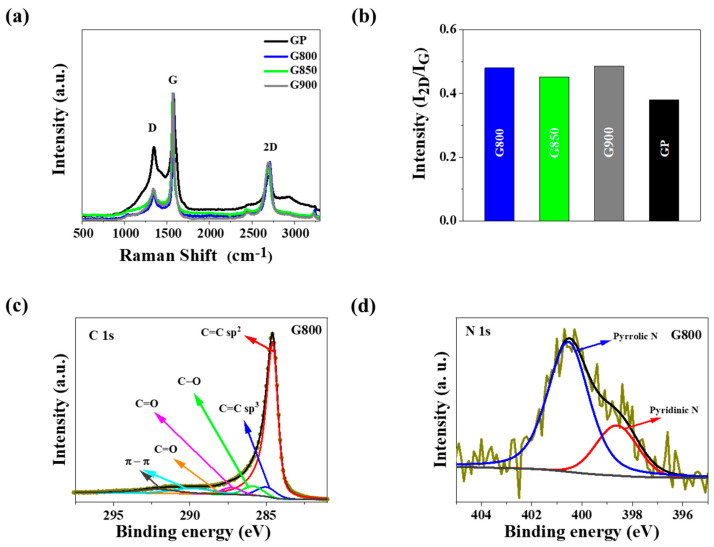
(**a**) Raman spectra and (**b**) intensity ratio of pristine graphite powder (GP) and N-doped exfoliated graphene obtained in GX series; (**c**) X-ray photoelectron spectroscopy (XPS) spectra of C1s and (**d**) N1s for G800 sample.

Analyses of Raman spectra for the GX series are presented in Figure 3a,b. The Raman spectra registered three characteristic bands for graphene D, G, and 2D, in which the Raman shift at the 532 nm laser line is around 1347 cm^−1^, 1577 cm^−1^, and 2697 cm^−1^, respectively [57]. The increase of the I_D_/I_G_ ratio indicated that the carbon matter became progressively more defective in the order: G800 (0.24), G850 (0.29), and G900 (0.30). With increasing carbonization temperature, a slight increase of the I_D_/I_G_ ratio is observed, which corresponds to a slightly lower graphitization degree of obtained materials. The 2D band is found to be a quantitative guide to distinguishing the layer number of FL-graphene [58,59,60]. As shown for the G800, G850, G900 samples and the graphite powder (GP) reference sample, the I_2D_/I_G_ ratio presented in Figure 3b is 0.49, 0.46, 0.49, and 0.38, respectively, which shows that the obtained materials are multi-layer graphene.

X-ray photoelectron spectroscopy (XPS) analysis was applied to determine the chemical composition of the materials’ surface. The XPS results are presented in Figure 3c,d, and Table 1. An example XPS C1s spectrum for the G800 sample exhibits different carbon bands; the main peaks can be ascribed to C−C sp^2^ (284.6 eV), C=C sp^3^ (285.0 eV), C−O (286.3 eV), C=O (287.7 eV), and O−C=O (288.6 eV) [61,62]. For all samples, a high total content of carbon was observed, ranging from 94.6 to 97.9 at.%. Such values are characteristic for pure graphene-based materials. 

Conversely, carbon matrixes obtained from *Chlorella vulgaris* are less rich in carbon, their carbon elemental content may rank from 61.6 to 84.6 wt.% [56]. The carbon content of the materials obtained is typical for graphene-based materials. This means that the obtained samples mainly consist of electroexfoliated graphene sheets, which largely determine the total carbon elemental content, while *Chlorella-vulgaris*-originated carbon makes a minor contribution. An example XPS N1s spectrum after deconvolution exhibits two characteristic bands placed at 398.7 and 400.5 eV, associated with pyridinic-N (N-6) and pyrrolic-N (N-5), respectively [61,63,64,65]. That proves that nitrogen in the graphene-based samples is exclusively bound in the form of these two functional groups. The presence of the two N-functional groups allows for improving the materials’ electrocatalytic properties. The overall nitrogen atomic content, as presented in Table 1, for the G800, G850, G900 samples was 1.6 at.% for G800, 2.2 at.% for G850, and 1.9 at.% for G900 samples. The O1s spectrum presented two bands with binding energy (BE) at 532.0 and 533.3 eV, which can be attributed to O=C–N in aromatic rings and C=O–C, respectively [61,66]. The total content of oxygen in G800, G850, and G900 samples was 6.9, 5.4, and 6.6 at.%, respectively.

### 3.2. Electroctrochemical Performance

**Figure 4:**
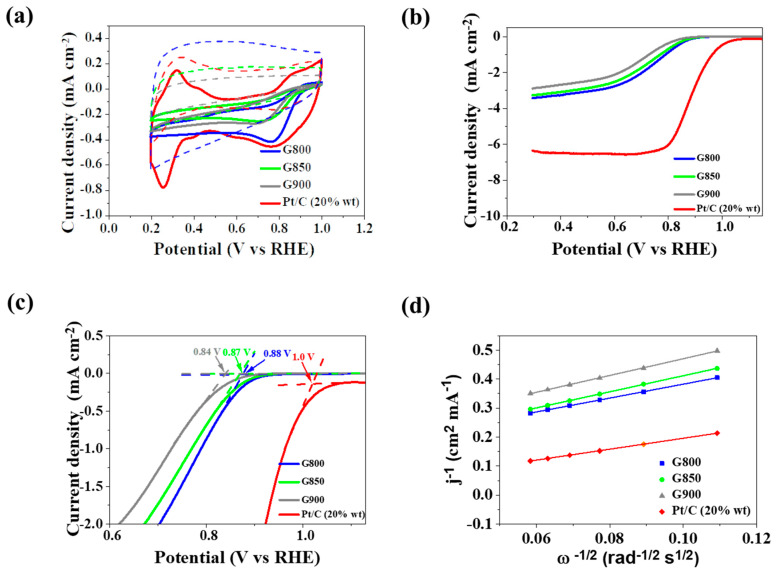
(**a**) Cyclic voltammetry (CV) curves recorded at 100 mV s^−1^ under N_2_ (dashed line) and O_2_ flow for the GX series and Pt/C catalyst; (**b**) Linear sweep voltammetry (LSV) curve of the prepared samples (10 mV s^−1^, 1600 rpm) for GX series and Pt/C catalyst; (**c**) Onset potential determination in expanded LSV curves; (**d**) Koutecky-Levich plot for the commercial Pt/C catalyst and GX series at +0.50 V.

Cyclic voltamperometry was the next step in the physicochemical characterization of the produced catalysts (CV), along with linear sweep voltamperometry to check catalytic activity in ORR. Catalytic ORR activity is fundamental for metal-air batteries, including Zn-air ones. In recent years, heteroatom-doped graphene has been one of the mainstream electrocatalysts, including ORR. Since carbonization temperature influences electric properties, such as the conductivity of a heat-treated material like *Chlorella vulgaris*, three catalysts were subjected to carbonization at 800 °C, 850 °C, and 900 °C. Such temperatures are usually high enough to convert many non-conductive organic materials into conductive carbon-based matrixes [52,67,68]. For comparison, another carbon-based material, a Pt/C (20 wt.% Pt) catalyst, was also tested under the same conditions. The tests were carried out in 0.1 M KOH electrolyte in an oxygen atmosphere. CV plots depicted in Figure 4a show characteristic cathodic peaks for G800, G850, and G900 placed at −0.23, −0.25, and −0.28 V vs. Ag/AgCl in the alkaline electrolyte saturated with oxygen, respectively. For the commercial Pt/C catalyst the cathodic peak was recorded at −0.28 V. Featureless voltammetric currents in the potential range of −1 V to 0 V were observed in a nitrogen atmosphere. This ascribes the origin to ORR and indicates good electrocatalytic properties in the GX sample series. Onset potential and the number of transferred electrons is very important in the evaluation of catalytic activity in ORR. Before calculating the value of this parameter, a linear sweep voltamperometry (LSV) was carried out. Figure 4b contains the LSV plots obtained at a rotation rate of 1600 rpm and a scanning rate of 5 mV s^−1^ in 0.1 M KOH in O_2_ saturated for N-doped graphene-based catalysts and the reference commercial Pt/C (20 wt.% Pt) catalyst. The G800 sample was characterized by the largest current density compared to the G850 and G900 samples, and the Pt/C catalyst. This result reveals that for these materials the preferred temperature for electrocatalytic activity in ORR is 800 °C. The values of the onset potential are presented in Figure 4c. For the samples G800, G850, and G900, they are 0.88 V, 0.87 V, and 0.84 V, respectively. The G800 sample exhibits the best catalytic properties, with the cathode peak at −0.2 V vs. Ag/AgCl, while the onset potential was 0.88 V. This is a relatively similar, though slightly smaller, value to commercial Pt/C catalyst. The obtained materials are based on graphene and are without metals; even though the onset potential is not satisfying, the number of transferred electrons described a desirable process in ORR. Analysis of the Koutecky-Levich (K-L) plot (Figure 4d) allowed us to determine the number of transferred electrons participating in the ORR reaction. LSV data in 0.5 V with a rotation speed in the range of 800 to 1600 rpm potential were used to create K-L curves. ORR for commercial Pt/C catalysts favors the direct four-electron pathway and the two-electron pathway for pristine graphene [69,70]. For the obtained N-doped graphene-based catalysts G800, G850, and G900, the number of transferred electrons is 3.78, 3.28, and 3.13, respectively. The *n* number signals that the four-electron transfer mechanism is dominant for the ORR. The high number of transferred electrons confirms that the acquired materials have similar properties to a commercial Pt/C catalyst. This, in turn, proves that these electrocatalysts, N-doped graphene, are both metal-free and potentially successful alternatives to a commercial Pt/C catalyst. N-functional groups in graphene structures are responsible for catalytic activities in ORR.

### 3.3. Rechargeable Zn-Air Battery Tests

Recently, due to cost and environmental issues, researchers have been undertaking intensive efforts to implement carbon-based materials as an alternative to noble metals like platinum. Nitrogen atoms are supposed to modulate electronic and surface properties of carbon matrixes to which they bond [71,72,73]. Some authors assume that N atoms, similarly to Pt, increase charge delocalization and the density of donor states near the Fermi level, while not compromising electronic conductivity [71,74,75]. After insertion, N atoms improve n-type conductivity of carbon matrixes and increase the electron transfer rates from the matrix to the adsorbed O_2_ molecules (upgrading the overall electrocatalytic activity) [71,75].

**Figure 5:**
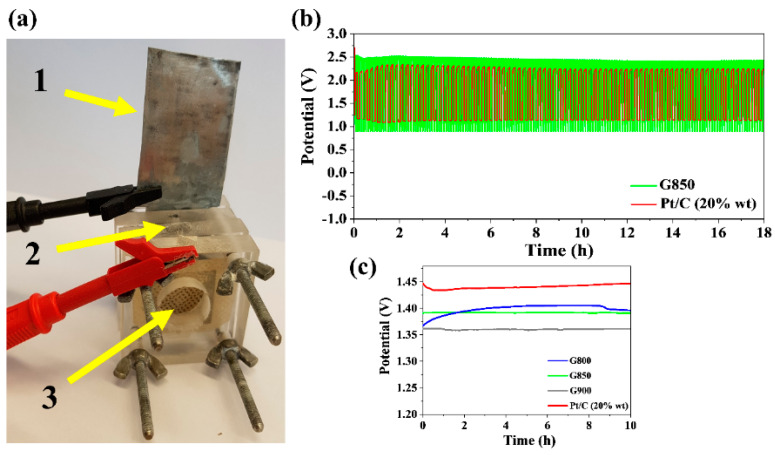
(**a**) Photograph of the home-made rechargeable Zn-air battery with an open circuit voltage of 1.39 V, where (1) Zn electrode, (2) electrolyte 6 M KOH with 0.2 M ZnCl_2_, (3) air electrode (GDL made of the catalyst under investigation); (**b**) galvanostatic charge/discharge cycling curves at 1 mA cm^−2^ of Zn-air batteries incorporating the G850 catalyst and the commercial Pt/C catalyst, respectively; (**c**) galvanostatic discharge curves at 1 mA cm^−2^ for G800, G850, G900 catalysts and the commercial Pt/C catalyst.

The ORR results of N-doped graphene-based catalysts indicate that these materials could be used as electrocatalysts in Zn-air batteries. For this purpose, the G800, G850, G900 samples were applied as cathodic catalysts as part of an air electrode, and were assembled into home-made rechargeable Zn-air batteries. The assembled batteries were typical two-electrode batteries with a zinc plate applied as anode. Figure 5a presents an example home-made battery setup for testing charge/discharge performance of the assembled Zn-air batteries. As has already been mentioned, a 6 M KOH solution with 0.2 M ZnCl_2_ was the electrolyte used in charge/discharge experiments. All Zn-air batteries using the G800, G850, and G900 catalysts were compared with similarly prepared Zn-air batteries with the Pt/C (20 wt.% Pt) catalyst used as the cathodic. After 250 cycles (each cycle consisted of two steps: 300 s of charge and 300 s of discharge), the results proved a very high battery stability regarding the charge/discharge potential range, as well as the potential of a fully charged battery. Similar behavior was noted for graphene-based catalysts and the Pt/C catalyst (Figure 5). However, the batteries that had graphene-based catalysts in a gas diffusion electrode offered a broader charge-discharge potential range, and a higher potential of a fully charged battery. These tests assumed that the N-functional groups in graphene-based catalysts are responsible for the promising catalytic performance resulting from ORR activity (cathode). The range of charge/discharge potential difference for batteries with N-doped graphene-based catalysts ranked from 2.5 V (charged) to 1 V (discharged). The open circuit potential (OCP) of Zn-air batteries with the G800, G850, G900 samples was stable and held potential for 10 h at 1.40, 1.39, and 1.36 V, respectively. The stability of OCP for N-doped graphene-based catalysts is very similar to the stability of OCP for a Pt/C catalyst (all OCP values close to 1.45 V). 

Lin et al. also investigated N/S doped porous carbons used as cathode material in Zn-air batteries working in 6 M KOH electrolyte. Beside several electrochemical tests, a homemade Zn-air battery was constructed and intensively tested in a cyclic charge-discharge experiment [27]. These batteries exhibited a very good cycling stability at 20 mAcm^−2^ for 12 h with a 10 min cycling period, i.e., no narrowing of the charge/discharge potential range was observed, nor was a decrease of the potential of a fully charged battery noted. The cycling test by Lin et al. was much shorter (12 h) than our cycling experiment (18 h). Thus, the current research offers considerable progress beyond the limit set by Lin et al. and others.

The synthesized N-doped graphene-based catalysts are a very good solution for the design of a metal-free electrocatalyst. Owing to the presence of nitrogen (N-5 and N-6 groups), the graphene-based catalysts are capable to perform ORR as effectively and intensively as Pt-loaded commercial catalysts. Promising electrochemical performance (nearly four-electron ORR mechanism and applicability in Zn-air batteries) was achieved at relatively low N doping levels, such as 2 at.% relative to the whole sample mass. It must be pointed out that N-doping occurred at carbon matrixes originated from *Chlorella vulgaris*, which underwent thermal degradation. Direct N-doping of graphene sheets is much less feasible since that sort of N-substitution occurs in much more severe experimental conditions [76,77]. HRTEM tests proved that this N-doped carbon matter was present as domains among well split graphene flakes (electroexfoliation).

## 4. Conclusions

In summary, efficiently working electrode materials were developed that could be used in Zn-air batteries with success. The synthesis process of N-doped graphene is low cost, environmentally friendly, and requires little effort. The combination of electroexfoliation, 3D structuring, and subsequent impregnation of graphene material with a *Chlorella vulgaris* suspension allows the introduction of pyridinic-N and pyrrolic-N nitrogen functional groups into the carbon material structure, as proven by XPS analysis. Despite the low nitrogen content of 1.8–2.2 at.%, the obtained materials exhibited catalytic activity in ORR. The transferred electron number (*n*) per O_2_ molecule in alkalic medium calculated by the K-L equation is 3.78 for the G800, 3.28 for the G850, and 3.13 for the G900 electrode; this is comparable to a reference Pt/C commercial catalyst despite the platinum making up 20% of the content by weight. Such a hybrid catalyst is expensive and has multiple components that require advanced recycling. Home-made Zn-air batteries constructed using the produced ORR catalysts (gas permeable cathode) exhibited very high stability during the charge/discharge process, comparable to the operating range of a rechargeable Zn-air battery employing a commercial Pt/C catalyst. The acquired metal-free, N-doped, and graphene-based catalysts are a promising class of electrode materials for electrochemical energy storage devices. High ORR activity was achieved for the relatively minor content of nitrogen, no more than 2 at.%. 

## Figures and Tables

**Table 1:** Chemical composition analyzed by XPS for the G800, G850, and G900 samples.

Sample	Binding Energy (eV)										
	284.6	285	286.3	287.7	288.6	289.6	292.1	532	533.3	398.7	400.5
	Elemental Content (at.%)										
	C							O		N	
G800	64.5	4.9	4.7	3.6	0.8	6.1	5.6	0.8	6.1	0.4	1.2
G850	63.1	7.8	4.9	3.5	1	5.6	5.5	1.1	4.3	0.8	1.4
G900	62.6	6.8	5.3	3.7	0.8	6.5	4.6	0.9	5.7	0.3	1.6
